# Proteomic analysis of meiosis and characterization of novel short open reading frames in the fission yeast *Schizosaccharomyces pombe*

**DOI:** 10.1080/15384101.2020.1779470

**Published:** 2020-06-17

**Authors:** Barbora Huraiova, Judit Kanovits, Silvia Bagelova Polakova, Lubos Cipak, Zsigmond Benko, Andrea Sevcovicova, Dorothea Anrather, Gustav Ammerer, Caia D. S. Duncan, Juan Mata, Juraj Gregan

**Affiliations:** a Department of Genetics, Faculty of Natural Sciences, Comenius University in Bratislava, Bratislava, Slovakia; b Department of Membrane Biochemistry, Inst. Of Animal Biochemistry and Genetics, Centre of Biosciences, Slovak Academy of Sciences, Bratislava, Slovakia; c Department of Genetics, Cancer Research Institute, Biomedical Research Center, Slovak Academy of Sciences, Bratislava, Slovakia; d Department of Molecular Biotechnology and Microbiology, Institute of Biotechnology, Faculty of Science and Technology, University of Debrecen, Debrecen, Hungary; e Mass Spectrometry Facility and Department of Biochemistry, Max Perutz Labs, University of Vienna, Vienna Biocenter, Austria; f Department of Biochemistry, University of Cambridge, Cambridge, UK; g Department of Chromosome Biology, Max Perutz Labs, Vienna Biocenter, University of Vienna, Vienna, Austria; h Advanced Microscopy Facility, Vienna Biocenter Core Facilities, Vienna, Austria

**Keywords:** Meiosis, fission yeast *Schizosaccharomyces pombe*, silac, short open reading frames

## Abstract

Meiosis is the process by which haploid gametes are produced from diploid precursor cells. We used stable isotope labeling by amino acids in cell culture (SILAC) to characterize the meiotic proteome in the fission yeast *Schizosaccharomyces pombe*. We compared relative levels of proteins extracted from cells harvested around meiosis I with those of meiosis II, and proteins from premeiotic S phase with the interval between meiotic divisions, when S phase is absent. Our proteome datasets revealed peptides corresponding to short open reading frames (sORFs) that have been previously identified by ribosome profiling as new translated regions. We verified expression of selected sORFs by Western blotting and analyzed the phenotype of deletion mutants. Our data provide a resource for studying meiosis that may help understand differences between meiosis I and meiosis II and how S phase is suppressed between the two meiotic divisions.

## Introduction

Sexual reproduction depends on meiosis, a process that generates haploid gametes from a diploid precursor cell. The fission yeast *Schizosaccharomyces pombe* is a useful model organism for studying meiosis. One of the advantages of using fission yeast is that highly synchronous meiosis can be induced by inactivation of the Pat1 protein kinase [1–4]. Moreover, a broad spectrum of genomic and proteomic tools is available. Progression of meiosis is accompanied by complex changes of gene expression [5]. These changes in fission yeast meiosis have been studied by various approaches including transcriptional profiling using DNA microarrays and ribosome profiling to investigate the translational landscape [6,7].

A comprehensive study analyzing changes of the *S. pombe* meiotic proteome using stable isotope labeling by amino acids in cell culture (SILAC) was published during the course of our work [8]. Krapp et al. quantified 3268 proteins throughout fission yeast meiosis induced by the inactivation of a temperature-sensitive allele of the Pat1 kinase (*pat1-114*) and found that the levels of 880 proteins changed at least 2-fold. Their study revealed a high degree of post-transcriptional regulation of protein levels and a global switch from anabolic to catabolic processes during meiosis [8].

In our current work, we performed SILAC based quantitative analysis of the *S. pombe* proteome during meiosis. In addition to *pat1-114*-induced meiosis, we used an improved synchronization protocol based on chemical inactivation of an ATP analog–sensitive form of the Pat1 kinase (*pat1-as2*), which eliminates negative effects of the higher temperature needed to inactivate the Pat1-114 kinase. We not only analyzed standard proteins, but also proteins encoded by short open reading frames (sORFs), which are usually defined as proteins smaller than 100 amino acids. Such sORFs were often ignored during genome annotations to minimize false positive ORFs [9–11]. However, recent analyses have revealed numerous examples of proteins encoded by sORFs that have important cellular functions [12,13]. We searched our SILAC based mass-spectrometry data and found peptides corresponding to novel sORFs that have been previously identified by ribosome profiling. We verified expression of selected sORFs by Western blot analysis and performed phenotypical characterization of deletion mutants. Finally, we discuss gene organization at the corresponding genomic regions and relevant refinements in the annotation of the fission yeast genome.

## Results and discussion

### SILAC based analysis of the meiotic proteome

There are several important differences between meiosis I (MI) and meiosis II (MII). Chiasma formation, mono-orientation of sister kinetochores, and protection of centromeric cohesion are key aspects of MI chromosomes that are absent during MII. In addition, MI is preceded by an S phase during which DNA is replicated but there is no S phase between MI and MII [14,15]. Quantitative comparison of the proteomes of various meiotic stages allows identification of proteins whose levels are differentially regulated. Such proteins that are specifically present or absent during a particular stage of meiosis may be important regulators of meiosis-specific processes. To identify such regulators, we used SILAC based proteome analysis to compare relative levels of proteins present during various stages of meiosis. SILAC labeling combined with high-resolution mass spectrometry is one of the key methods for quantitative proteomics [16] that is also available for fission yeast [8,17].

**Figure 1.**
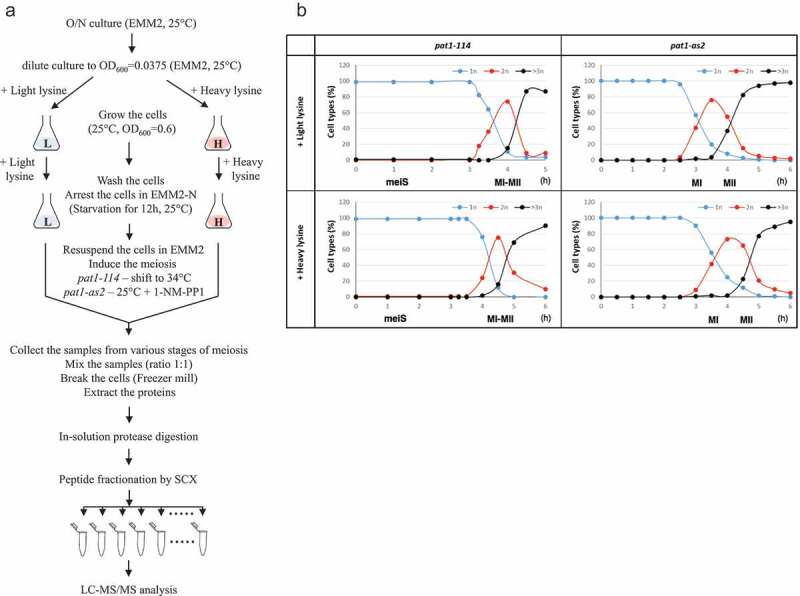
Flowchart of the SILAC based proteome analysis and progression of *pat1-*induced meiosis.

We used SILAC based proteome analysis in synchronous meiotic cultures induced by inactivation of a temperature-sensitive allele *pat1-114* to compare relative levels of proteins present during premeiotic S phase (meiS) with the interval between meiotic nuclear divisions (MI-II), when S phase is absent (Figure 1, Table S1). Although this synchronization protocol based on the inactivation of a temperature-sensitive allele of the Pat1 kinase (*pat1-114*) has been widely used to study meiosis in the fission yeast *S. pombe*, it is not ideal for studying meiotic divisions because of chromosome missegregation defect [18,19]. Previous studies showed that *pat1-114*-induced meiosis differs from wild-type meiosis in some aspects, such as chromosome segregation. Whereas in wild-type cells sister centromeres segregate to the same pole in anaphase I, in meiosis induced by inactivation of Pat1-114 by elevated temperature sister centromeres segregate to the same pole very inefficiently in anaphase I cells [18,19]. In order to overcome this obstacle, we have developed a synchronization protocol based on *pat1-as2*. Chemical inactivation of an ATP analog–sensitive form of the Pat1 kinase (*pat1-as2*) by adding the ATP analog 1-NM-PP1 allows the induction of synchronous meiosis without the need of elevated temperature. In *pat1-as2*-induced meiosis, chromosomes segregate with higher fidelity and spore viability is higher than in *pat1-114* meiosis [2–4]. We used *pat1-as2*-induced meiotic cultures to compare relative levels of proteins extracted from cells harvested around MI with those of MII (Figure 1, Table S1).

(a) Unlabeled (+ light lysine) and heavy lysine (+ heavy lysine) labeled diploid *S. pombe* cells were arrested by nitrogen starvation and meiosis was induced by shifting the cells to 34°C (*pat1-114*) or by adding 1-NM-PP1 (*pat1-as2*). Unlabeled and heavy lysine labeled cells from various stages of meiosis were mixed in equal amounts and protein extracts were prepared. After digestion with lysC protease, peptides were fractionated by strong cation exchange (SCX) and analyzed by mass spectrometry (LC-MS/MS). (b) Meiotic cells as described in (a) were fixed, stained with DAPI and nuclei were counted in 100 cells per time point. Shown are the fractions of cells that contained one nucleus (1n), two nuclei (2n) or more than two nuclei (3n or more) at the indicated time points after meiosis induction (hours). MeiS, MI-MII, MI and MII indicate when cells were harvested for the SILAC based proteome analysis.

To exclude possible isotope effects of the heavy ^13^C_6_ lysine and differences between batches of labeled amino acids, we performed experimental replicates with reversed labels. Unlabeled meiS was analyzed with heavy lysine labeled MI-MII transition (meiS (L) + MI-MII (H)) and heavy lysine labeled meiS was analyzed with unlabeled MI-MII transition (meiS (H) + MI-MII (L)). Similarly, unlabeled MI was analyzed with heavy lysine labeled MII (MI (L) + MII (H)) and heavy lysine labeled MI was analyzed with unlabeled MII (MI (H) + MII (L)) (Table S1). Normalized ratios (heavy/light) of peptides corresponding to selected proteins involved in DNA replication and chromosome segregation are shown in Table 1.

**Table 1.** Normalized ratios (heavy/light) of peptides corresponding to selected proteins involved in DNA replication and chromosome segregation. Nda2 and Act1 are used as controls.

**Proteins involved in DNA replication**	**MI-MII (heavy lysine)/meiS (light lysine)**	**meiS (heavy lysine)/MI-MII (light lysine)**
Pcn1 (proliferating cell nuclear antigen PCNA)	0,91	1,10
Cdc45 (DNA replication pre-initiation complex subunit)	0,94	1,11
Mcm4 (MCM replicative helicase complex subunit)	0,97	1,02
Mcm7 (MCM replicative helicase complex subunit)	0,70	1,68
Pol1 (DNA polymerase alpha catalytic subunit)	0,78	1,37
Mrc1 (mediator of replication checkpoint)	0,16	3,41
Tos4 (FHA-containing DNA binding protein)	0,07	6,02
Dfp1 (Hsk1-Dfp1 kinase complex regulatory subunit)	8,98	0,16
Nda2 (alpha tubulin)	1,06	0,92
Act1 (actin)	0,98	0,99
**Proteins involved in chromosome segregation**	**MII (heavy lysine)/** **MI (light lysine)**	**MI (heavy lysine)/MII (light lysine)**
Top2 (DNA topoisomerase II)	0,97	1,10
Sgo2 (shugoshin)	0,95	0,94
Mad1 (spindle assembly checkpoint protein)	1,01	1,09
Cnd2 (condensin complex subunit)	1,43	0,76
Rec8 (cohesin complex subunit)	0,13	5,10
Spo4 (protein kinase)	14,90	0,11
Nda2 (alpha tubulin)	1,01	0,95
Act1 (actin)	1,00	0,96

### Verification of proteins encoded by sORFs identified by ribosome profiling

**Figure 2.**
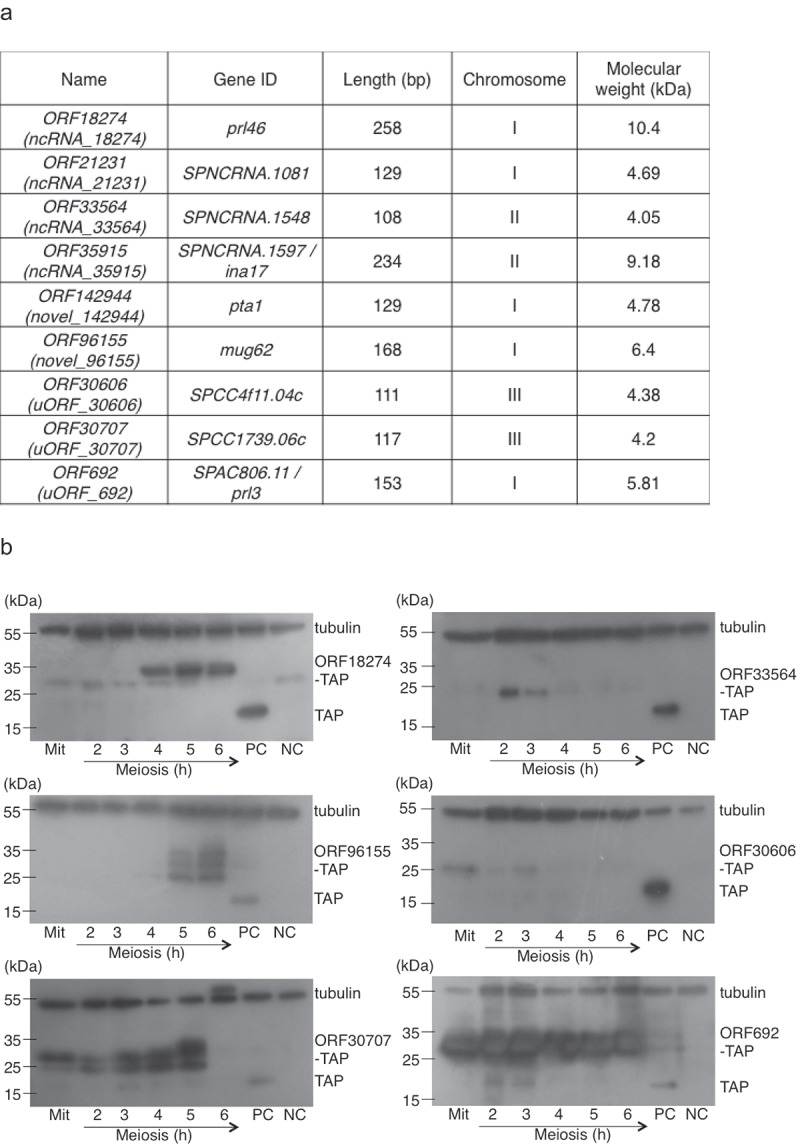
Verification of proteins encoded by novel sORFs identified by ribosome profiling.

Ribosome profiling of *S. pombe* diploid cells undergoing meiosis identified 373 sORFs encoding short proteins that were at least 30 amino acids long that have not been previously described (Table S2) [7]. These included short proteins in ncRNAs, unannotated regions and 5ʹ-UTRs (encoded in uORFs). We searched our SILAC based mass-spectrometry results for peptides corresponding to these 373 novel sORFs identified by ribosome profiling and found unique peptides corresponding to nine sORFs (Figure 2(a), Figure S1, Table S3).

(a) List of sORFs for which corresponding peptides were identified by mass-spectrometry. (b) *pat1-114* cells expressing indicated TAP-tagged proteins were arrested by nitrogen starvation and released into meiosis at 34°C (Figure S2). Cells were harvested at the indicated time points (hours) after meiosis induction and protein extracts were analyzed by Western blotting. Protein extracts were also prepared from cycling vegetative cells (Mit). As a positive control (PC), protein extracts were prepared from a pool of cells expressing TAP tag alone, harvested at 4, 5 and 6 hours after meiosis induction. As a negative control (NC), protein extracts were prepared from a pool of wild-type cells, harvested 2–6 hours after meiosis induction. TAP tag was detected using rabbit antiperoxidase antibody linked to peroxidase and tubulin was detected using mouse-anti-α-tubulin antibody. Molecular weight marker (kDa) is indicated on the left. Additional Western blots are shown in Figure S3.

Next, we constructed strains expressing C-terminally TAP-tagged ORF18274, ORF33564, ORF96155, ORF30606, ORF30707 and ORF692. Western blot analyses revealed bands of expected sizes but also additional bands (Figure 2(b), Figure S2, Figure S3). Further experiments are needed to clarify what these additional bands represent. While all six TAP-tagged proteins were detected in meiotic extracts, ORF30606-TAP, ORF30707-TAP and ORF692-TAP were present also in extracts from vegetative cells (Figure 2(b), Figure S2, Figure S3). ORF18274-TAP (Prl46-TAP) and ORF692-TAP (Prl3-TAP) were independently constructed and detected by Western blotting by Duncan and Mata [7]. However, we noticed that ORF18274-TAP is larger than originally described [7]. This is probably due to extended N-terminus, which starts already before the beginning of the non-coding RNA *prl46*.

During the course of this work, there were changes in annotations of three sORFs [11]. *ORF35915* has been annotated as the second exon of the *ina17* gene [20] and *ORF96155* as the first exon of the *mug62 *gene [7]. Detailed analysis of the sequence variants identified an indel error that affected the gene structure annotation of *pta1*, whose coding sequence was extended at the 3ʹ-end and included *ORF142944* [21]. Thus, it is likely that *ORF35915, ORF142944* and *ORF96155* do not encode independent short proteins but they are part of larger genes (Figure S1).

### Phenotypical characterization of sORFs deletion mutants

We analyzed the consequences of deleting *ORF18274, ORF35915, ORF142944, ORF30707* and *ORF692*. In a haploid *S. pombe* strain, we were able to delete *ORF18274, ORF35915, ORF30707, ORF692* but not *ORF142944*. Tetrad analysis of a diploid strain heterozygous for *ORF142944* deletion showed that spores carrying *ORF142944* deletion germinated but did not form colonies (data not shown). This result is consistent with the finding that *ORF142944* is part of the *pta1 *gene, which is essential for cell growth [10,21].

**Figure 3.**
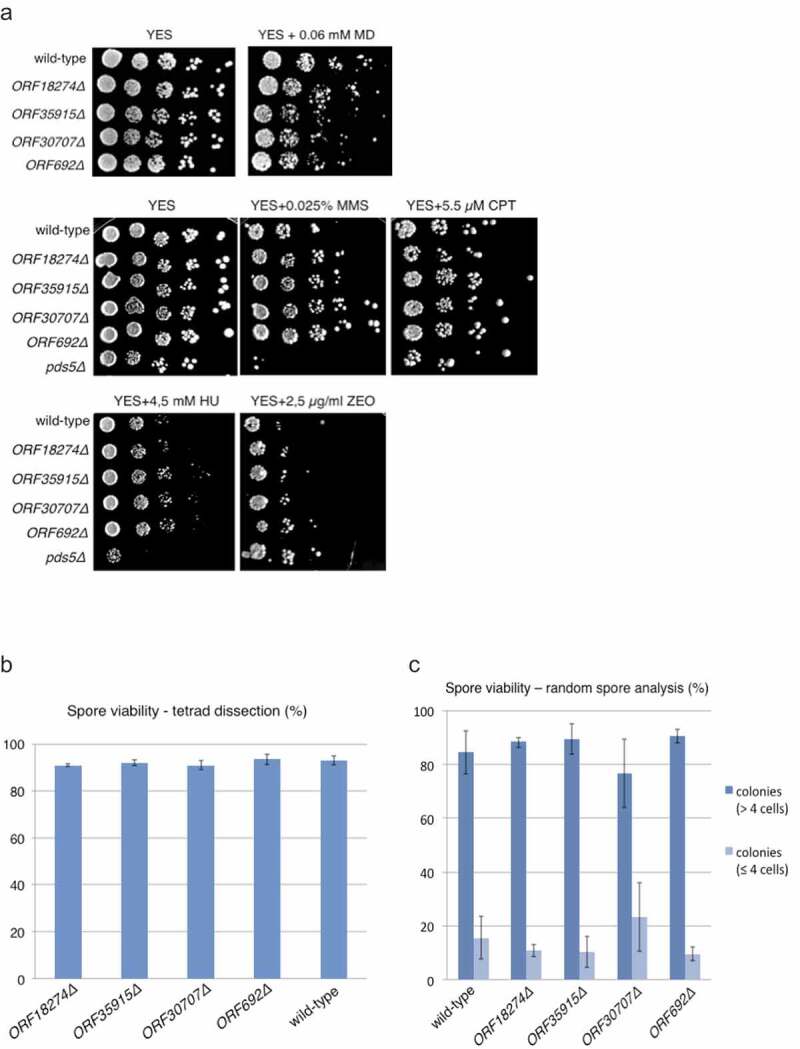
*ORF18274Δ, ORF35915Δ, ORF30707Δ* and *ORF692Δ* cells are not sensitive to DNA damaging agents and produce viable spores.

We next analyzed phenotypes of *ORF18274Δ, ORF35915Δ, ORF30707Δ* and *ORF692Δ* deletion strains. Mutant vegetative cells showed no apparent growth defects or altered cell morphology (data not shown). The growth of mutant cells was similar to wild type in the presence of DNA damaging agents such as methyl methanesulfonate, camptothecin, hydroxyurea, zeocin and menadione (Figure 3(a)). Chromosome segregation in mutant cells, as scored by GFP labeled centromere of chromosome I, was similar to wild type during both mitosis and meiosis (data not shown). Spore viability in mutant strains was similar to wild type, suggesting that there is no major meiotic defect in mutant cells (Figure 3(b,c)). We conclude that *ORF18274, ORF35915, ORF30707* and *ORF692* are dispensable for vegetative growth under all tested conditions and production of viable spores.

(a) Cells were grown on YES medium for one day, diluted and spotted onto YES plates containing the indicated amounts of menadione (MD), methyl methanesulfonate (MMS), camptothecin (CPT), hydroxyurea (HU) or zeocin (ZEO). Plates were incubated for 3 days at 32°C. *pds5Δ* was used as a control that is known to be sensitive to DNA damaging agents. (b) Spore viability of the indicated strains was measured by tetrad dissection in two independent experiments. 80 tetrads were dissected for each strain. (c) Spore viability of the indicated strains was measured by random spore analysis. 100,000 spores were plated per plate, incubated for 30 hours at 32°C and 200 spores/colonies were scored in at least two independent experiments. Microcolonies consisting of up to four cells and colonies containing more than four cells were scored.

Taken together, we performed SILAC based quantitative analysis of the *S. pombe* proteome during meiosis. Our results provide a resource for studying meiosis that may help understand differences between MI and MII and how S phase is suppressed between the two meiotic divisions. Our meiotic proteome datasets revealed unique peptides corresponding to only nine sORFs, out of 373 sORFs that have been previously identified by ribosome profiling as new translated regions. It is possible that more of these short proteins are present in meiotic and/or vegetative *S. pombe* cells, however their reliable detection will require more sensitive analyses including enrichment for short proteins before the mass-spectrometry analysis. Our results are consistent with previous findings that *ORF35915, ORF142944* and *ORF96155* do not encode independent short proteins but they are part of larger genes. They are also consistent with the notion that *ORF21231, ORF18274, ORF33564, ORF30606, ORF30707* and *ORF692* encode short proteins. However, we cannot exclude the possibility that these six sORFs are also part of larger genes. While we detected ORF21231 only by mass-spectrometry, ORF18274, ORF33564, ORF30606, ORF30707 and ORF692 were detected by both mass-spectrometry and Western blotting. The role of these short proteins remains unknown. Future experiments should include detailed analyses of mutant phenotypes and sensitive *in silico* searches to assess possible conservation of identified sORFs during evolution. Identification of sORFs that encode proteins and deciphering their roles are important future goals arising from our current work and other proteomic and ribosome profiling studies.

## Materials and methods

### Strain construction

We constructed TAP-tagging plasmids containing long regions homologous to the target gene according to our protocol described in Cipak et al. [22] for all nine sORFs (*ORF18274*, *ORF21231*, *ORF33564*, *ORF35915*, *ORF142944*, *ORF96155*, *ORF30606*, *ORF30707* and *ORF692*). We transformed these plasmids into a haploid *S. pombe* strain JG12017 and verified successful tagging in yeast transformants by PCR. We constructed strains expressing C-terminally TAP-tagged ORF18274, ORF33564, ORF96155, ORF30606, ORF30707 and ORF692 but not ORF142944, where no yeast transformants were obtained. Western blot analyses revealed bands of expected sizes but also additional bands (Figure 2(b), Figure S2, Figure S3). No bands were observed in extracts prepared from strains carrying ORF21231-TAP and ORF35915-TAP (data not shown).

Genotypes of strains and the figures and tables in which each was used are in Table S4. Genes were deleted as described in Gregan et al. [23]. Spore viability was determined as described in Phadnis et al. [24].

### Meiotic synchronization and SILAC labeling

Diploid *S. pombe* strains carrying temperature sensitive *pat1-114* (JG16328) or ATP analog-sensitive *pat1-as2* (JG16419) were incubated at 25°C over-night in EMM2 liquid medium (3.0 g/l potassium hydrogen phthalate, 2.2 g/l Na_2_HPO_4_, 5.0 g/l NH_4_Cl, 1.0% (w/v) glucose, 75 mg/l lysine, supplemented with salts, vitamins and minerals) [2,3]. The cells were collected by centrifugation, diluted in fresh EMM2 medium supplemented with 75 mg/l lysine (light sample) or 75 mg/l heavy lysine (heavy sample) into OD_600_ = 0.0375 and grown at 25°C until OD_600_ = 0.5–0.6. After centrifugation the cells were washed 3 times with deionized water, resuspended in EMM2-N medium (3.0 g/l potassium hydrogen phthalate, 2.2 g/l Na_2_HPO_4_, 1.0% (w/v) glucose, supplemented with salts, vitamins and minerals) and incubated at 25°C for 12 h to arrest the cells in G_1_ phase. Arrested cells were centrifuged and resuspended in the same volume of EMM2 medium supplemented with 75 mg/l lysine (light sample) or 75 mg/l heavy lysine (heavy lysine labeled sample). Meiosis was induced by shifting the cells to 34°C (*pat1-114*) or by adding 1-NM-PP1 (Toronto Research Chemicals) to 25 μM and incubated at 25°C (*pat1-as2*). Unlabeled and heavy lysine labeled cells from various stages of meiosis were collected by filtration through 0.45 μm membrane disc filter (Pall Corporation). Cells were frozen in liquid nitrogen and disrupted by Cryogenic Grinder (6775 Freezer/Mill Cryogenic Grinder, SPEX SamplePrep).

Heavy lysine was purchased from Cambridge Isotope Laboratories (U-13C6, CLM-2247-0.25), TRIzole Reagent from Invitrogen (15,596–026, 100 ml), GN-6 Metricel MCE Membrane Disc Filters from Pall Corporation (66,265, 47 mm, plain, sterile) and Magnetic Filter Funnels from Pall Corporation (4242, 47 mm, 300 mL capacity).

### Protein extraction

Yeast powders from light sample (0.1 g) and heavy lysine labeled sample (0.1 g) isolated from particular stages of meiosis were mixed and resuspended in 6 ml of TRIzol reagent. Proteins were extracted by vigorous shaking at 4°C for 15 min. The sample was centrifuged at 12,000 g for 15 min at 4°C to remove insoluble material. Supernatant was incubated for 5 min at RT, extracted with chloroform (ratio TRIzol to chloroform was 5:1) and centrifuged at 12,000 g for 15 min at 4°C. Organic phase containing DNA and proteins was collected and DNA was precipitated by mixing the supernatant with 1.8 ml 100% ethanol and centrifuged at 2000 g for 5 min at 4°C. Phenol-ethanol supernatant was collected and mixed with 9 ml of isopropanol to precipitate the proteins. The proteins were collected by centrifugation at 12,000 g for 10 min at 4°C and washed 3 times by 0.3 M guanidine hydrochloride in 95% ethanol and 1 time in 95% ethanol. Vacuum dried proteins were dissolved in 1 ml of 8 M urea supplemented with 0.5 M NH_4_HCO_3_ for 1 h at RT.

### Mass spectrometry analysis

Protein extracts were reduced with DTT and then alkylated with iodoacetamide. Protein solution was diluted with water to 6 M urea and then digested with LysC (Wako) at 1:30 ratio at 37°C overnight. The digests were desalted and lyophilized, then dissolved and chromatographically separated on a strong cationic exchanger (SCX) with a mixed salt- and pH-gradient in 15% acetonitrile (ACN). Up to 70 fractions were collected and ACN was removed by sample concentration in the speed vac. The peptide fractions were separated in a second dimension on a C18 column on a nano HPLC (Dionex, Thermo Scientific) applying 1 hour gradient. Eluting peptides were analyzed on a QExactive Orbitrap (Thermo Scientific) in a data-dependent mode. The 12 most intense peptides in the survey scan recorded at 70,000 resolution at 200 m/z, were subjected to CID fragmentation with 30% collision energy. CID spectra were recorded at 17,500 resolution and an AGC target value of 5E4. The MS data were searched with MaxQuant 1.4 [25]. against the *S. pombe* reference database (https://www.pombase.org, 2013–03-19) and the sequences of the sORFs (Table S2) with the following settings: LysC specificity, carabamidomethylation on Cys as fixed, oxidation of methionin and acetylation of protein N-termini as variable modification. The SILAC quan node was selected with ^13^C_6_ lysine as the heavy label. All other parameters were set to default. Results were filtered on protein and peptide level for a 1% FDR.

### Western blot analysis

Proteins were separated by electrophoresis through 12% polyacrylamide gels containing SDS (0.1%) and transferred to a PVDF membrane (Immobilon-P membrane with 0.45 μm pore size from Millipore). The membrane was blocked with 2% (w/v) milk-PBS-T (phosphate buffer saline buffer with 0.1% (v/v) Tween-20) and probed with antibodies. TAP-tagged proteins were detected using rabbit antiperoxidase antibody linked to peroxidase (PAP, Dako; 1:10,000 dilution) in 0.1% PBS-T (137 mM NaCl, 2.7 mM KCl, 10 mM Na_2_HPO_4_, 1.8 mM KH_2_PO_4_, 0.1% (v/v) Tween-20). Tubulin was detected using mouse-anti-α-tubulin antibody (Sigma-Aldrich T5168; 1:10,000 dilution) and rabbit anti-mouse IgG-HRP secondary antibody (Santa Cruz Biotechnology; 1:5000 dilution) in 2% (w/v) milk PBS-T.

## Supplementary Material

Supplemental Material
