# Supplementary material for: Proteomic analysis of meiosis and characterization of novel short open reading frames in the fission yeast Schizosaccharomyces pombe
Source: Cell Cycle. 2020 Jun 17;19(14):1777–85. doi: 10.1080/15384101.2020.1779470 (PMC7469465; doi:10.1080/15384101.2020.1779470)
Supplement: Supplemental Material [file KCCY_A_1779470_SM4665.zip › Supplementary information/Supplemental_Figures.pdf]

ORF33564

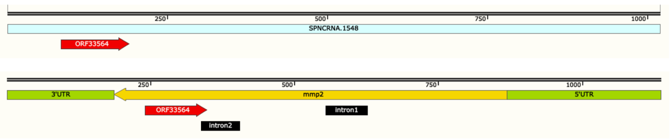

ORF35915

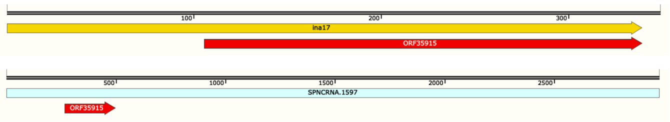

ORF142944

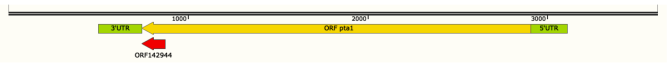

ORF96155

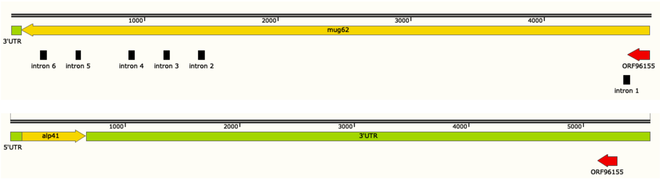

ORF30606

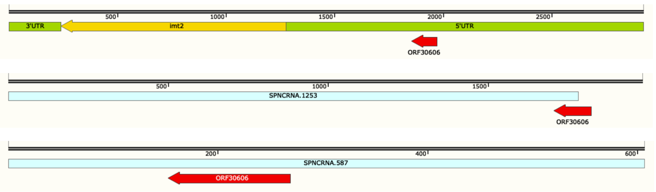

ORF30707

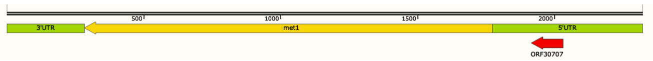

ORF692

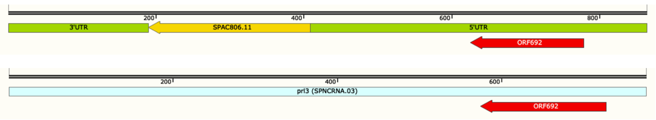

Figure S1. Annotated chromosomal regions with studied sORFs.

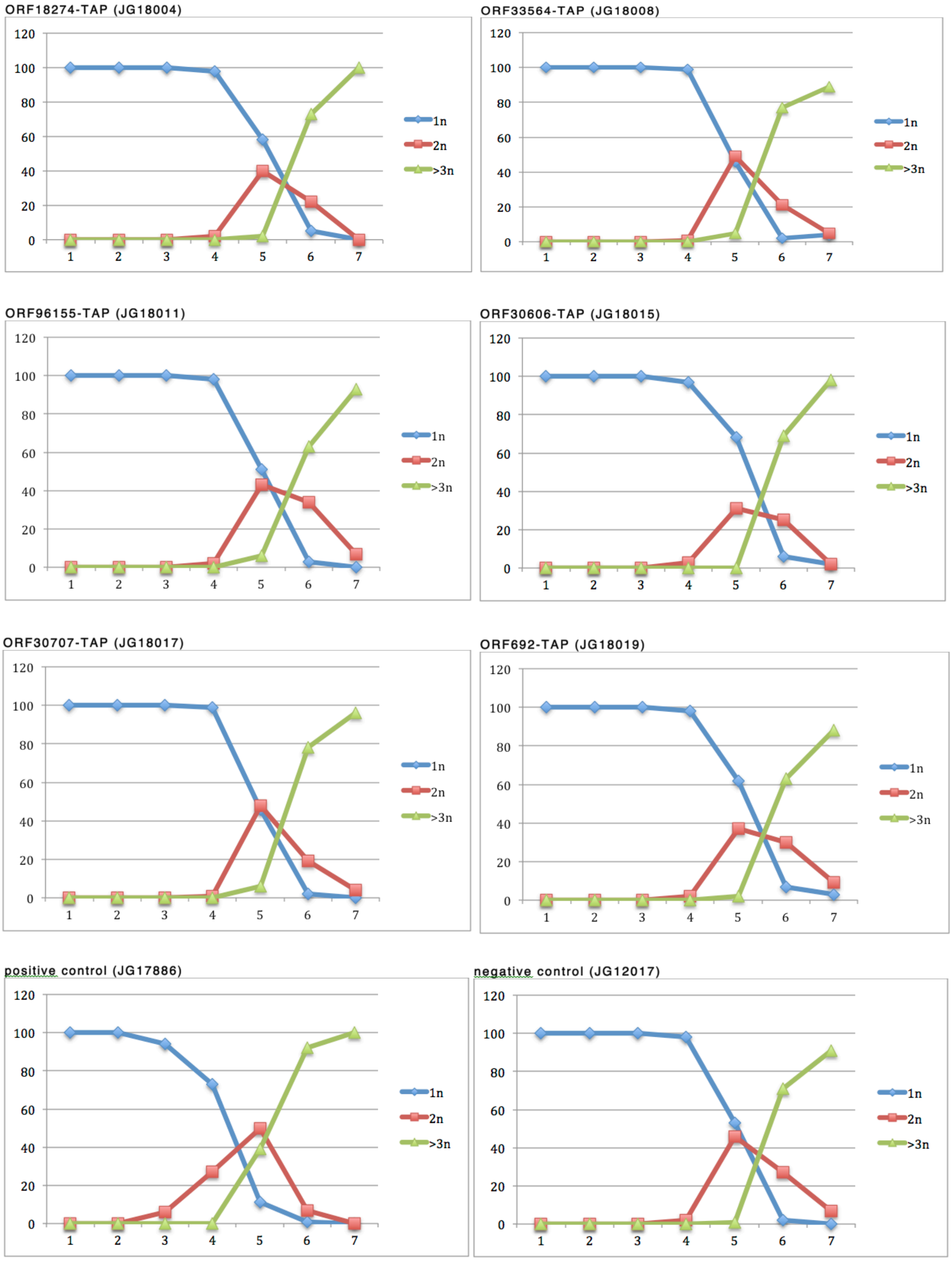

Figure S2. Progression of *pat1-114* cells expressing indicated TAP-tagged proteins into meiosis.

pat1-114 cells expressing indicated TAP-tagged proteins were arrested by nitrogen starvation and released into meiosis at 34°C. Fixed cells were stained with DAPI and nuclei were counted in 100 cells per time point. Shown are the fractions of cells that contained one nucleus (1n), two nuclei (2n) or more than two nuclei (3n or more) at the indicated time points after meiosis induction (hours). The progression of meiosis in all tested mutant strains was similar to that in wild-type cells.

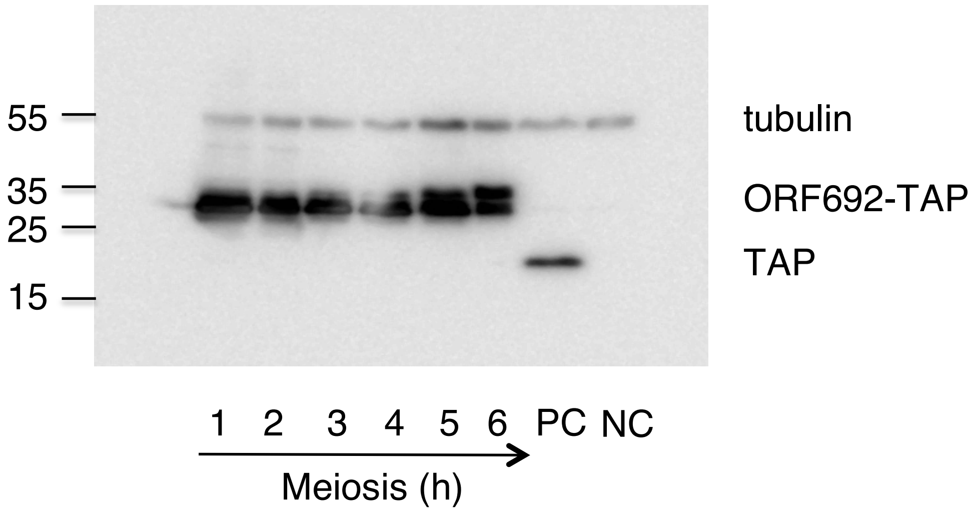

Figure S3. Western blot analysis of ORF692-TAP.
Protein extracts were prepared and analyzed by Western blotting as described in Figure 2B.
