# Supplementary material for: Proteomic analysis of meiosis and characterization of novel short open reading frames in the fission yeast Schizosaccharomyces pombe
Source: Cell Cycle. 2020 Jun 17;19(14):1777–85. doi: 10.1080/15384101.2020.1779470 (PMC7469465; doi:10.1080/15384101.2020.1779470)
Supplement: Supplemental Material [file KCCY_A_1779470_SM4665.zip › Supplementary information/Table1.docx]

Table 1. Normalized ratios (heavy/light) of peptides corresponding to selected proteins involved in DNA replication and chromosome segregation. Nda2 and Act1 are used as controls.

| **Proteins involved in DNA replication** | **MI-MII (heavy lysine) / meiS (light lysine)** | **meiS (heavy lysine) / MI-MII (light lysine)** |
| --- | --- | --- |
| Pcn1 (proliferating cell nuclear antigen PCNA) | 0,91 | 1,10 |
| Cdc45 (DNA replication pre-initiation complex subunit) | 0,94 | 1,11 |
| Mcm4 (MCM replicative helicase complex subunit) | 0,97 | 1,02 |
| Mcm7 (MCM replicative helicase complex subunit) | 0,70 | 1,68 |
| Pol1 (DNA polymerase alpha catalytic subunit) | 0,78 | 1,37 |
| Mrc1 (mediator of replication checkpoint) | 0,16 | 3,41 |
| Tos4 (FHA-containing DNA binding protein) | 0,07 | 6,02 |
| Dfp1 (Hsk1-Dfp1 kinase complex regulatory subunit) | 8,98 | 0,16 |
| Nda2 (alpha tubulin) | 1,06 | 0,92 |
| Act1 (actin) | 0,98 | 0,99 |
| **Proteins involved in chromosome segregation** | **MII (heavy lysine) /**  **MI (light lysine)** | **MI (heavy lysine) / MII (light lysine)** |
| Top2 (DNA topoisomerase II) | 0,97 | 1,10 |
| Sgo2 (shugoshin) | 0,95 | 0,94 |
| Mad1 (spindle assembly checkpoint protein) | 1,01 | 1,09 |
| Cnd2 (condensin complex subunit) | 1,43 | 0,76 |
| Rec8 (cohesin complex subunit) | 0,13 | 5,10 |
| Spo4 (protein kinase) | 14,90 | 0,11 |
| Nda2 (alpha tubulin) | 1,01 | 0,95 |
| Act1 (actin) | 1,00 | 0,96 |
