# Supplementary material for: Proteomic analysis of meiosis and characterization of novel short open reading frames in the fission yeast Schizosaccharomyces pombe
Source: Cell Cycle. 2020 Jun 17;19(14):1777–85. doi: 10.1080/15384101.2020.1779470 (PMC7469465; doi:10.1080/15384101.2020.1779470)
Supplement: Supplemental Material [file KCCY_A_1779470_SM4665.zip › Supplementary information/TableS4.docx]

**Table S4. *S. pombe* strains**

| Strain | Genotype | Used in Figure/Table |
| --- | --- | --- |
| JG16328 | *h-/h- lys1-131/lys1-131 ade6-M210/ade6-M216 pat1–114/pat1–114 lys1::BleoMX-mat-Pc* | Figure 1, Table S1, Table S3 |
| JG16419 | *h-/h- lys1-131/lys1-131 ade6-M210/ade6-M216 pat1::NatMX/pat1::NatMX pat1-as2(L95A)::HygMX/pat1-as2(L95A)::HygMX lys1::BleoMX-mat-Pc* | Figure 1, Table S1, Table S3 |
| JG12017 | *h- pat1-114 ade6-210* | Figure 2B (negative control), Figure S2, Figure S3 (NC) |
| JG17886 | *h+/h+ ade6-M210/ade-M216 pat1-114/pat1-114 meu5::TAP-kanMX/pat1-114 meu5::TAP-kanMX* | Figure 2B (positive control), Figure S2, Figure S3 (PC) |
| JG18004 | *h- pat1-114 ade6-M210 ORF18274::TAP-KanMX* | Figure 2B, Figure S2 |
| JG18008 | *h- pat1-114 ade6-M210 ORF33564::TAP-KanMX* | Figure 2B, Figure S2 |
| JG18011 | *h- pat1-114 ade6-M210 ORF96155::TAP-KanMX* | Figure 2B, Figure S2 |
| JG18015 | *h- pat1-114 ade6-M210 ORF30606::TAP-KanMX* | Figure 2B, Figure S2 |
| JG18017 | *h- pat1-114 ade6-M210 ORF30707::TAP-KanMX* | Figure 2B, Figure S2 |
| JG18019 | *h- pat1-114 ade6-M210 ORF692::TAP-KanMX* | Figure 2B, Figure S2, Figure S3 |
| JG18152 | *h^90^ dh1L::lacOp-ura4+ his7::NatMX lacI-GFP ORF18274::KanMX* | Figure 3 |
| JG18153 | *h^90^ dh1L::lacOp-ura4+ his7::NatMX lacI-GFP ORF35915::KanMX* | Figure 3 |
| JG18157 | *h^90^ dh1L::lacOp-ura4+ his7::NatMX lacI-GFP ORF30707::KanMX* | Figure 3 |
| JG18159 | *h^90^ dh1L::lacOp-ura4+ his7::NatMX lacI-GFP ORF692::KanMX* | Figure 3 |
| JG18161 | *h^90^ dh1L::lacOp-ura4+ his7::NatMX lacI-GFP* | Figure 3 |
| JG18192 | *h^90^  ade6-M216 ura4-D18 leu1-32 pds5::KanMX* | Figure 3 |

Other auxotrophic markers were not scored, sORFs are described according to Duncan and Mata, 2014.
